# Sex Differences in Incidence and Outcome of Out-of-Hospital Cardiac Arrest Within a Local Health Network

**DOI:** 10.3389/fcvm.2022.870696

**Published:** 2022-04-08

**Authors:** Melanie R. Wittwer, Emily Aldridge, Cindy Hein, Mel Thorrowgood, Chris Zeitz, John F. Beltrame, Margaret A. Arstall

**Affiliations:** ^1^Adelaide Medical School, University of Adelaide, Adelaide, SA, Australia; ^2^Northern Adelaide Local Health Network, Adelaide, SA, Australia; ^3^College of Medicine and Public Health, Flinders University, Adelaide, SA, Australia; ^4^SA Ambulance Service, Eastwood, SA, Australia; ^5^Central Adelaide Local Health Network, Adelaide, SA, Australia

**Keywords:** out-of-hospital cardiac arrest, sex, gender, outcomes - health care, aetiology (etiology), socioeconomic status, epidemiology

## Abstract

**Introduction:**

Sex and gender differences in presentation and characteristics of out-of-hospital cardiac arrest (OHCA) are established in cohorts with presumed cardiac aetiology but not non-cardiac etiology. This study investigated the effect of sex on incidence and outcome of OHCA according to presumed and adjudicated aetiology within a local health network.

**Methods:**

Population-based observational cohort study of emergency medical services (EMS) attended OHCAs within an Australian local health network. Cases identified from an EMS registry between 2012-2016 were linked to a hospital registry. Age-standardised incidence and baseline characteristics were stratified by sex for EMS-treated OHCA, non-EMS witnessed presumed cardiac and obvious non-cardiac sub-cohorts, and hospitalised cases. Logistic regression was used to explore the primary outcome of survival to hospital discharge.

**Results:**

We identified 2,024 EMS-attended and 780 EMS-treated OHCAs. The non-EMS witnessed sub-cohorts comprised 504 presumed cardiac and 168 obvious non-cardiac OHCAs. Adjudicated aetiology was recorded in 123 hospitalised cases. Age-standardised incidence for women was almost half that of men across all groups. Across cohorts, women were generally older and arrested with a non-shockable initial rhythm in an area of low socioeconomic status. There was no sex difference in the primary outcome for the main EMS-treated cohort or in the non-cardiac sub-cohorts. The sex difference in outcome in the presumed cardiac sub-cohort was not present after multivariable adjustment.

**Conclusions:**

There are sex differences in incidence and outcome of EMS-treated OHCA that appear to be driven by differences in susceptibility to cardiac arrhythmias and underlying etiology, rather than treatment delays or disparities.

## Introduction

Incidence, characteristics, and outcomes of out-of-hospital cardiac arrest (OHCA) differ according to sex. Women represent around 40% of the OHCA population attended by emergency medical services (EMS) but present with fewer established predictors of survival including increased age, unwitnessed arrest, arrest within a private residence, and non-shockable initial rhythm compared with men (1, 2). Precipitating non-cardiac aetiology leading to OHCA, as confirmed by diagnostic testing or autopsy, is also more common in women than men, and is associated with fewer survival predictors, such as shockable initial rhythm, and poor overall survival (3–9). Nonetheless, sex differences in outcomes have not been investigated according to adjudicated cardiac and non-cardiac etiology. Recent meta-analyses found that adult women were up to 50% less likely to survive to hospital discharge or 30 days after OHCA compared with men (2, 10). Adjusting for known survival predictors fully accounts for observed sex differences in survival to hospital discharge in Australian and international populations (1, 11–14). It is likely that the high rates of non-cardiac aetiology and associated non-shockable initial rhythm in women play a key role in driving the relationship with poor outcome after OHCA, but this area remains under-researched. Socioeconomic status (SES) is another important determinant of cardiovascular health, particularly in women (15, 16). Low SES is associated with a high incidence of OHCA and poor survival (17); however, limited studies suggest that low SES is associated with poor survival in men but not women (18, 19).

The primary study objective was to investigate the effect of sex on survival to discharge in a cohort of EMS-treated OHCAs and sub-cohorts of non-EMS-witnessed presumed cardiac and obvious non-cardiac cases. The secondary objectives were to report incidence stratified by age and sex, explore the effect of SES on survival according to sex, and to investigate sex differences in adjudicated aetiology in the sub-cohort transported to hospital.

## Methods

### Study Design

This was a retrospective observational study of all adult OHCAs within the Northern Adelaide Local Health Network (NALHN), South Australia. The study cohorts were generated by linking an EMS-based and a hospital-based OHCA registry for all cases occurring within a NALHN catchment as defined by postcode. The STROBE (Strengthening the Reporting of Observational Studies in Epidemiology) guidelines (20) were followed and the Central Adelaide Local Health Network Human Research Ethics Committee approved the study [HREC/15/TQEH/89].

### Study Setting

The SA Ambulance Service (SAAS) provides a two-tier EMS where patients are treated by paramedics on scene across the state of South Australia (SA). NALHN comprises two public hospitals that service a population of 395,000 across 631 km^2^ within the northern metropolitan area of Adelaide, SA. Compared with the rest of Australia, both SA and NALHN are characterised by low SES and are ranked in the 37th and 19th percentiles according to the Index of Relative Socio-Economic Advantage and Disadvantage (IRSAD), respectively (21). SAAS and NALHN hospitals follow the ANZCOR resuscitation guidelines (22). Cardiac catheterisation and targeted temperature management are performed at the discretion of treating clinicians according to local guidelines.

### Data Sources and Definitions

The SAAS Cardiac Arrest Registry (SAAS-CAR), described previously (23), was searched from 2012–16 for all cases aged ≥18 years within NALHN using the postcode associated with arrest location. Patients without attempted resuscitation by EMS had high rates of missing data (85 missing for initial rhythm, 63 witness status, 63% bystander CPR) and were included for incidence rate calculations only. The main cohort comprised all EMS-treated OHCAs including obvious non-cardiac aetiologies such as trauma, asphyxia, exsanguination, overdose etc., while the sub-cohorts comprised EMS-treated, non-EMS-witnessed OHCA with (presumed cardiac) or without obvious non-cardiac cause. Attempted resuscitation was defined as any chest compressions or any defibrillation by paramedics. Arrest location (e.g., private residence) and response times were not available due to limitations in data capture within the study period. The primary outcome of survival to hospital discharge was extracted for cases transported to non-NALHN hospitals.

The Northern Adelaide Local Health Network (NALHN) OHCA registry is a hospital-based quality assurance initiative (24). Variables are obtained from linkage with existing clinical registries and abstraction from the hospital medical record. Ethnicity was frequently documented as unknown and therefore excluded from this analysis. The hospitalised sub-cohort was formed by manually linking cases with the NALHN OHCA registry using age, sex, arrest date, and time of call.

The 2011 IRSAD was generated from census data by the Australian Bureau of Statistics (abs.gov.au) according to postal area code (POA) and linked to postcode of arrest. Although residential postcodes better reflect individual SES, they were not available for analysis. Higher national deciles indicate low levels of disadvantage and high levels of advantage.

### Outcomes

The primary outcome was survival to hospital discharge. Secondary outcomes included incidence per 100,000 person-years, whether the patient was transported to hospital (excluding patients transferred for certification of death), and survival with good neurological recovery (cerebral performance category, CPC, 1-2) in hospitalised patients.

### Statistical Analysis

Crude and age-standardised incidence rates per 100,000 person-years were explored according to sex for EMS-attended OHCAs with attempted resuscitation aged ≤ 20 years, to match with available population data. To account for dynamic changes in the at-risk population over the study period, enumerated NALHN population data (Australian Bureau of Statistics, compiled and presented by.id) was averaged between data available for 2011 and 2016 (25). Adjusted rates were calculated using the direct method across 5-year age groups from 20 to >85 years and applied to the 2001 Australian standard population. Age was missing, presumed at random, in eight cases so an inflation factor was calculated as the percentage of missing data and applied to both crude and age-adjusted incidence rates (Supplementary Table 1).

Descriptive statistics were used to explore differences between males and females in all cohorts. Comparisons between sexes were performed using Wilcoxon Sum Rank Tests, Chi-Squared Tests or Fisher's Exact Tests as appropriate for skewed continuous and categorical variables.

Exploratory binary logistic regressions investigated the association between sex and survival to hospital discharge for both the main cohort and presumed cardiac sub-cohort, while adjusting for available survival predictors (age, witness status, bystander CPR, and shockable rhythm). The obvious non-cardiac sub-cohort was too small and survival rate too low to permit multivariable analysis. Interactions between sex and each covariate were tested in the adjusted models and removed if insignificant. Odds ratios (OR), 95% confidence intervals (95%CI), and comparison and global *P*-value are presented.

*P*-values less than or equal to 0.05 were regarded as significant and adjustments were not made for multiple comparisons. Analyses were performed using SPSS 26 (IBM SPSS Statistics, Armonk, NY, USA).

## Results

**Figure 1:**
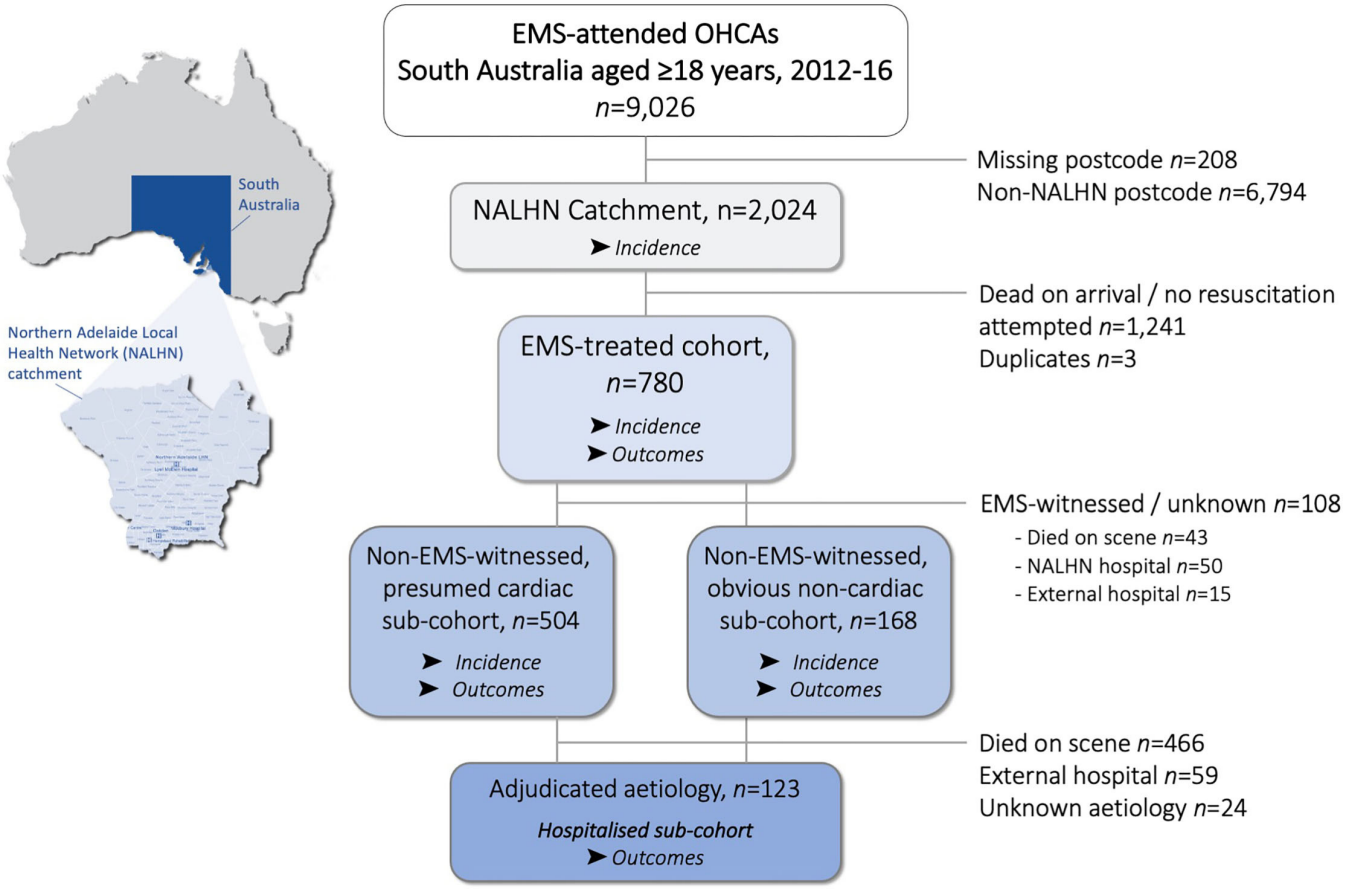
Flowchart of out-of-hospital cardiac arrests (OHCA) aged ≥18 years occurring within the Northern Adelaide Local Health Network (NALHN) catchment area in South Australia, Australia from 2012–2016.

There were 9,026 EMS-attended cardiac arrests aged ≥18 years identified from SAAS-CAR between 2012–16, of which 2,024 (23%) occurred within a NALHN postcode and 780 were EMS-treated (Figure 1). There was no difference in proportion of males vs. females receiving attempted resuscitation (38% vs. 39% of all attended arrests, *p* > 0.05). In the sub-cohorts of non-EMS witnessed cases, 504 were of presumed cardiac origin and 168 were of obvious non-cardiac origin. The hospitalised sub-cohort consisted of 123 cases with adjudicated aetiology documented in the NALHN OHCA registry, excluding 24 with unknown etiology.

**Figure 2:**
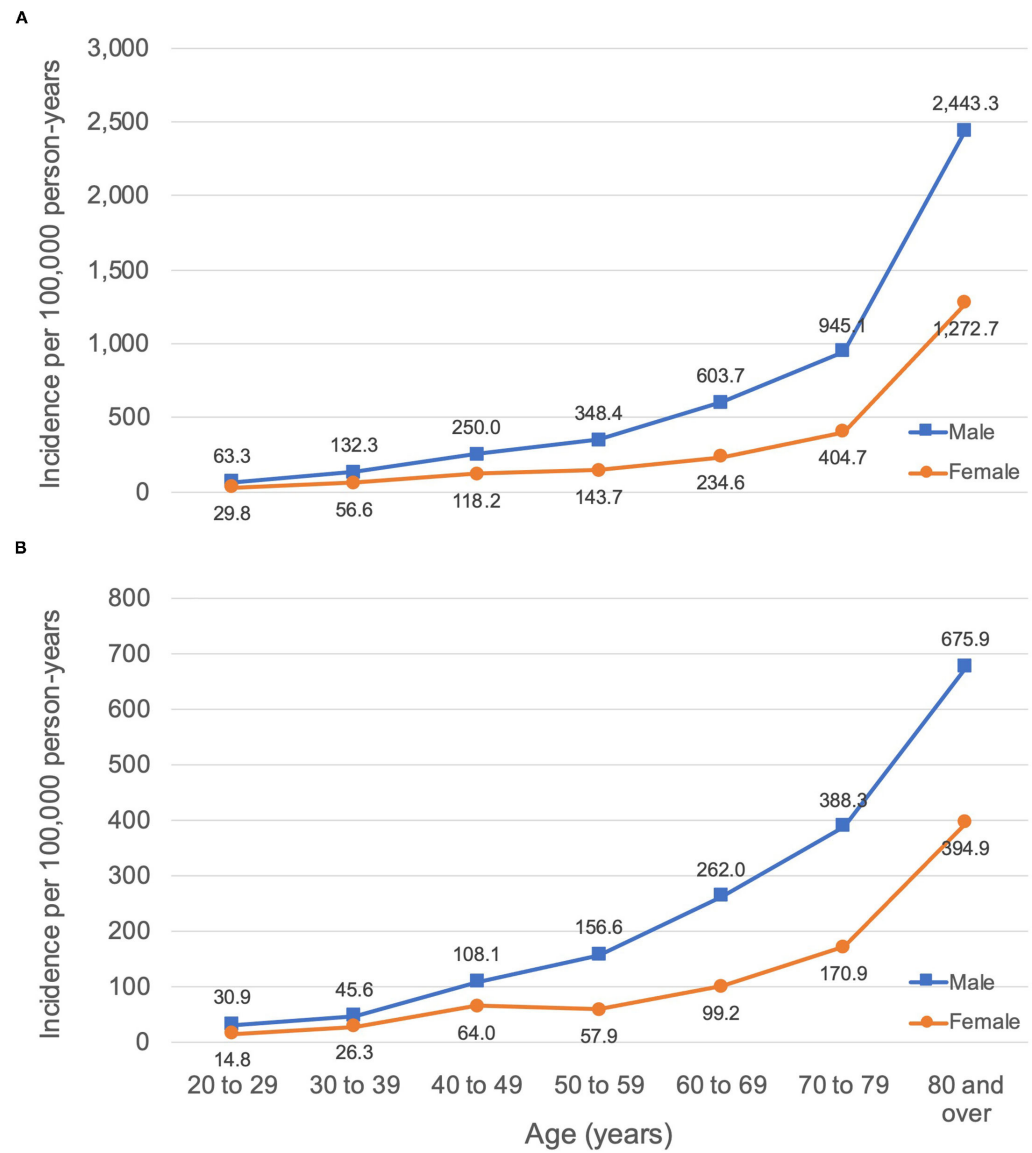
Incidence of OHCA per 100,000 person-years according to age and sex within a local health network in **(A)** EMS-attended OHCA, **(B)** EMS-treated OHCA.

Crude and age-adjusted incidence rates of OHCAs aged ≥20 years according to sex are presented in Table 1. Incidence in women was similar to that of men 10–20 years younger for EMS-attended and EMS-treated OHCAs (Figure 2).

**Table 1:** Incidence of OHCA aged ≥20 years within NALHN according to sex, 2012–2016.

	**Total**	**Females**	**Male**
EMS-attended^*^	***n*** **= 1,970**	***n*** **= 691**	***n*** **= 1,279**
Crude	148.7	100.3	199.6
Age-standardised	139.9	96.0	184.3
EMS-treated cohort^*^	***n*** **= 772**	***n*** **= 273**	***n*** **= 499**
Crude	57.3	39.2	76.4
Age-standardised	54.6	38.1	71.8
**Non-EMS witnessed presumed cardiac sub-cohort**	***n*** **= 501**	***n*** **= 163**	***n*** **= 338**
Crude	36.8	23.4	50.9
Age-standardised	34.7	22.8	47.2
Non-EMS witnessed obvious non-cardiac sub-cohort^*^	***n*** **= 161**	***n*** **= 63**	***n*** **= 98**
Crude	12.3	9.0	15.8
Age-standardised	12.2	9.0	15.6

*Data is presented per 100,000 person-years*.

* *Inflation factor applied to crude and age-standardised incidence rates, excepting female EMS-treated rates and non-EMS witnessed obvious non-cardiac rates for females*.

### EMS-Treated Cohorts

Sex differences in characteristics of the main adult EMS-treated OHCA cohort and sub-cohorts are presented in Table 2. Women represented 35% of the main cohort, 33% of the presumed cardiac sub-cohort, and 38% of the non-cardiac sub-cohort, were a median 4–6 years older than men on presentation, and had similar rates of presumed cardiac diagnosis as men. Women in the main cohort and presumed cardiac sub-cohort, but not the obvious non-cardiac sub-cohort were less likely to present with VF/VT and more likely to present with asystole than men. OHCA was more likely to occur in an area associated with higher levels of disadvantage (lowest 5 deciles) in women than men in the presumed cardiac sub-cohort, but this difference was not observed for the main or non-cardiac cohorts.

**Table 2:** Characteristics of EMS-treated OHCAs within NALHN according to sex.

	**EMS-treated cohort** ***n*** **= 780**			**Non-EMS witnessed sub-cohorts**					
				**Presumed cardiac** ***n*** **= 504**			**Obvious non-cardiac** ***n*** **= 168**		
**Characteristics**	**Sex**		**Missing**	**Sex**		**Missing**	**Sex**		**Missing**
	**Female** *n* **= 273**	**Male** *n* **= 507**		**Female** *n* **= 165**	**Male** *n* **= 339**		**Female** *n* **= 63**	**Male** *n* **= 105**	
Age	68 [49–82]	64 [50–76]^*^	8 (1%)	72 [53-82]	67 [56-77]	–	53 [42-71]	47 [35-65]	–
IRSAD decile ≤ 5	205 (75%)	377 (74%)	–	134 (81%)	247 (73%)^*^	–	43 (67%)	83 (79%)	–
Witnessed			4 (0.5%)			–			–
EMS-witnessed	42 (15%)	61 (12%)	–	–	–	–	–	–	–
Bystander	102 (38%)	221 (44%)	–	77 (47%)	117 (52%)	–	25 (39%)	44 (42%)	–
Unwitnessed	127 (47%)	223 (44%)	–	88 (53%)	222 (48%)	–	39 (61%)	61 (58%)	–
Bystander CPR	148 (56%)	291 (59%)	28 (3.7%)	107 (66%)	217 (65%)	7 (1.4%)	39 (61%)	71 (69%)	
Initial rhythm			8 (1%)			3 (0.6%)			1 (0.6%)
VF/VT	50 (19%)	158 (32%)^*^	–	33 (21%)	137 (41%)^*^		5 (8%)	5 (5%)	–
PEA	79 (29%)	220 (44%)	–	38 (23%)	61 (18%)		21 (33%)	31 (30%)	–
Asystole	139 (52%)	126 (25%)^*^	–	92 (56%)	140 (41%)^*^		37 (59%)	68 (65%)	–
Presumed cardiac	192 (71%)	385 (76%)	1 (0.1%)	165 (100%)	339 (100%)	–	0 (0%)	0 (0%)	–
NALHN Hospital	71/92 (77%)	126/178 (71%)		34/42 (81%)	87/119 (73%)	–	13/20 (65%)	13/25 (52%)	–
Transported to hospital	92 (34%)	178 (35%)		42 (25%)	119 (35%)^*^	–	20 (31%)	25 (24%)	–
Survived to discharge	24 (9%)	65 (13%)	8 (1%)	12 (7%)	50 (15%)^*^	1 (0.2%)	4 (6%)	3 (3%)	2 (1.2%)

*Data presented as median [interquartile range] and number (percentage)*.

* *P-value <0.05; p-values reflect data that excludes missing values*.

*CPR, cardiopulmonary resuscitation; EMS, emergency medical services; IRSAD, Index of relative social advantage and disadvantage; NALHN, Northern Adelaide Local Health Network; PEA, pulseless electrical activity; VF, ventricular fibrillation; VT, ventricular tachycardia*.

There was no significant sex difference in unadjusted survival to hospital discharge observed in the main cohort (9% women vs. 13% men; OR: 0.66, 95% CI: 0.40-1.08, *p* = 0.099). Exploratory analyses were performed and an interaction between sex and shockable rhythm, but not sex and age, SES, or other predictors, was observed. On multivariable analysis, higher odds of survival were associated with shockable rhythm in both males and females, decreasing age, bystander witness, and EMS witness, as well as IRSAD deciles, such that for every increase in IRSAD decile the odds of survival increased by 11% (Table 3). There was no difference in survival from hospital arrival to discharge in all cases transported to hospital, including non-NALHN hospitals (women 29% vs. men 42%, *p* = 0.14).

**Table 3:** Multivariable logistic regression model: predictors of survival to hospital discharge after EMS-treated OHCA (main cohort), *n* = 751.

**Characteristics**	**Comparison**	**Odds Ratio (95% CI)**	***p*** **-value**
Female sex	Shockable vs. non-shockable	1.26 (1.09–1.46)^*^	0.001
Male sex	Shockable vs. non-shockable	1.46 (1.30–1.65)	<0.001
Age, per *y*		0.98 (0.96-1.00)	0.012
IRSAD decile		1.11 (1.00–1.23)	0.041
Bystander witnessed vs. unwitnessed		3.00 (1.56–5.77)	<0.001
EMS witnessed vs. unwitnessed		6.77 (2.97–15.5)	<0.001
Presumed cardiac cause		0.83 (0.39–1.76)	0.621

* *Interaction p < 0.05*.

*EMS, emergency medical services; IRSAD, Index of relative social advantage and disadvantage*.

In unadjusted analyses of the presumed cardiac sub-cohort, women were less likely than men to survive to hospital discharge (7% women vs. 15% men; OR: 0.45, 95%CI 0.23–0.87, *p* = 0.018). No interactions were observed between sex and age, SES, or other predictors. Multivariable analysis revealed that sex was not associated with higher odds of survival to hospital discharge (OR: 0.76, 95% CI 0.35–1.64, *p* = 0.48), nor was bystander CPR (OR: 1.02, 95% CI 0.49-2.11, *p* = 0.96). Decreasing age (OR: 0.97, 95% CI 0.95–0.99, *p* = 0.013), bystander witness (OR: 3.04, 95% CI 1.47–6.27, *p* = 0.003), shockable rhythm (OR: 16.1, 95% CI 6.99-37.0, p < 0.001), and increasing IRSAD deciles (OR: 1.13, 95%CI: 1.00, 1.28, *p* = 0.046) were associated with higher odds of survival to hospital discharge.

### Hospital-Treated Sub-cohort

Sex differences in survival to hospital discharge were explored according to adjudicated aetiology (cardiac vs. non-cardiac, excluding unknown) in a small sub-cohort of non-EMS witnessed OHCAs transported to NALHN hospitals (Table 4). Cardiac aetiology represented 68% of known adjudicated diagnoses (57% including unknown diagnoses) and was significantly more prevalent in men than women (76% vs. 50%, *p* = 0.01). Women with cardiac aetiology were younger than men, but there were no other statistically significant sex differences in arrest characteristics or outcomes within groups. In cases with a pre-hospital presumed cardiac diagnosis, precipitating aetiology was confirmed as cardiac in fewer women than men when cases with unknown diagnoses were included (53% vs. 75%, *p* = 0.029).

**Table 4:** Characteristics of EMS-treated, non-EMS witnessed OHCAs treated at NALHN hospital according to adjudicated aetiology and sex, *n* = 123.

	**Cardiac** ***n*** **= 84**		**Non-cardiac** ***n*** **= 39**	
**Characteristics**	**Female *n* = 18**	**Male *n* = 66**	**Female *n* = 18**	**Male *n* = 21**
Age	51 [41–65]	65 [54–72]^*^	53 [43–70]	50 [34–67]
IRSAD decile ≤ 5	14 (78%)	47 (71%)	11 (61%)	15 (71%)
Bystander witnessed	11 (61%)	53 (80%)	8 (44%)	11 (52%)
Bystander CPR	15 (83%)	47 (71%)	13 (72%)	16 (76%)
**Initial rhythm**				
VF/VT	14 (78%)	56 (85%)	1 (6%)	2 (10%)
PEA	0 (0%)	3 (5%)	7 (39%)	5 (24%)
Asystole	4 (22%)	7 (11%)	10 (56%)	14 (67%)
Pre-hospital presumed cardiac diagnosis	18 (100%)	65 (98%)	7 (39%)	12 (57%)
GCS 3 on arrival	15 (83%)	49/64 (77%)	16 (89%)	20 (95%)
Sustained ROSC	16 (89%)	61 (92%)	18 (100%)	20 (95%)
ST-elevation	6/16 (38%)	25/60 (42%)	1/16 (6%)	4/19 (21%)
Inpatient admission	16 (89%)	59 (89%)	15 (83%)	19 (90%)
Survived to discharge	7 (39%)	37 (56%)	4 (22%)	1 (5%)
Neurological recovery (CPC 1-2) at discharge	7 (39%)	35/65 (54%)	4 (22%)	1 (5%)
12-month survival	7 (39%)	35 (53%)	4 (22%)	1 (5%)

*Data presented as median [interquartile range] and number (percentage)*.

* *P-value < 0.05; p-values reflect data that excludes missing values*.

*CPR, cardiopulmonary resuscitation; EMS, emergency medical services; IRSAD, Index of relative social advantage and disadvantage; NALHN, Northern Adelaide Local Health Network; PEA, pulseless electrical activity; VF, ventricular fibrillation; VT, ventricular tachycardia*.

## Discussion

We report sex differences in incidence and outcome of consecutive EMS-attended and -treated OHCA within a local health network. Within these populations, women were almost half as likely to experience OHCA compared with men after age-standardisation. Although women in the sub-cohort with non-EMS-witnessed presumed cardiac OHCA were less likely to survive to hospital discharge than men in unadjusted analyses, this association was not present in the adjusted model. Exploratory analyses highlighted the discrepancy between presumed and adjudicated aetiologies and pointed to a survival advantage for hospitalised women with adjudicated non-cardiac aetiology.

### Sex Differences in Incidence

Few studies have reported sex differences in age-standardised incidence of EMS-attended and EMS-treated adult OHCAs, irrespective of etiology. Unadjusted and age-adjusted rates stratified by sex were consistent with comparable previous studies, confirming that men consistently experience OHCA at a rate more than double that of women (13, 26–28). Similar to the delayed onset of cardiovascular disease in women, our data and that of others suggests that the incidence of OHCA in women of any given age group is similar to that of men 10 years younger (27–29).

### Sex Differences in Survival

Women in the presumed cardiac sub-cohort were 55% less likely to survive to hospital discharge than men in unadjusted analyses. Once adjusted for available predictors of survival that differ between males and females (age, SES, witness status, and initial rhythm) the sex difference in outcome disappeared. It is likely that a smaller magnitude of difference in outcome between sexes exists for the main cohort of EMS-treated OHCAs, but the sample may not have been sufficiently powered. Only a few studies have reported sex differences in outcome of all-cause OHCA with the survival rate for men ranging from 1 to 5.5% higher than women (13, 30–32). Attenuation of the magnitude of difference in outcome between sexes may be due to the inclusion of obvious non-cardiac etiologies such as asphyxia, exsanguination, and overdose, the outcomes of which may not differ between males and females. Although we found that outcomes were similar between sexes in the small non-cardiac sub-cohort, this hypothesis has only been investigated in one other study of patients presenting with shockable rhythm and requires further validation (33). Previous reports of sex differences in survival appear contradictory; however, all of the larger OHCA registries (*n* > 10,000) report unadjusted survival and favourable neurological prognosis at hospital discharge as consistently higher in men than women, with no difference (1, 11–14, 30) or even a favouring of women (34) after adjustment, irrespective of differences in population subsets. The observed sex differences in survival across our cohorts were explained by the older age of women, their higher rate of arrest in a low SES area with an initial non-shockable rhythm, and lower likelihood of a confirmed cardiac aetiology than men. Our results confirm a different distribution of risk factors, such as age and SES, and precipitating etiologies between sexes rather than a male survival advantage.

### Interaction Between Sex and Established Predictors of Survival

There were no significant interactions in adjusted models between sex and age, bystander witness, or bystander CPR, respectively. Similar to Bray et al. (1) our findings did not show increased survival in younger Australian women. We did not observe any sex differences in pre-hospital treatment such as bystander CPR or EMS resuscitation, which is in contrast to some previous studies (1, 35). In the main EMS-treated cohort, but not the presumed cardiac sub-cohort, we observed a significant interaction between sex and initial rhythm where the relationship between shockable rhythm and survival was stronger in men than women. Although women are 50% less likely to present with a shockable rhythm after adjustment for established predictors of survival, (27, 30, 36) we again confirm that non-shockable initial rhythm predicts poor outcome regardless of sex (1, 12, 30, 34, 37). Poor survival in women is therefore directly related to their lower incidence of shockable initial rhythm, which, in our population, is likely due to sex differences in susceptibility to cardiac arrhythmias and underlying aetiology (38, 39), rather than treatment delays or disparities.

### Effect of Socioeconomic Status on Survival

Consistent with international studies, we found that SES was a predictor of survival after OHCA in adjusted analyses (40, 41). Each increase in SES decile (more advantaged) was associated with an 11% increase in odds of survival to hospital discharge after EMS-treated OHCA (adjusted OR: 1.11, 95% CI 1.00–1.23). Women with a presumed cardiac OHCA were more likely to arrest in a postcode associated with low SES but this was not the case for the full cohort that included obvious non-cardiac etiologies such as asphyxia, exsanguination, and overdose. However, the interaction between sex and SES was not significant and differences in survival rate across low and high SES did not vary between men and women. Wells et al. (18) found no interaction between sex and individual-level education or occupation in a cohort of EMS-treated non-traumatic OHCAs with shockable initial rhythm. Similarly, Jonsson et al. (19) reported no interaction between sex and area-level income and area-level education in all EMS-treated OHCAs, excluding EMS-witnessed. These findings are somewhat surprising given that a stronger association between low SES risk of cardiac arrest and sudden cardiac death has been observed in women compared with men, even after adjustment for traditional risk factors (42). Importantly, our results should be considered as hypothesis-generating only as the study population is biased and over-representative of low SES (IRSAD ≤ 5 in 75% of the study population). The importance of SES in determining outcome of OHCA has been highlighted in this study and should be explored in larger state-wide and national analyses.

### Sex Differences According to Adjudicated Etiology

Cause of arrest documented by EMS providers does not reflect true aetiology in many cases and these discrepancies may contribute to observed differences in outcome between sexes (43). We performed an in-depth exploration of aetiology as documented in the hospital medical record or autopsy report for the hospitalised sub-cohort. The sample was underpowered to detect a significant difference in outcome and should be considered as hypothesis generating only. The results suggest that survival after adjudicated cardiac OHCA is higher in men, whereas survival after non-cardiac OHCA is higher in women. Only 53% of hospitalised women with a pre-hospital presumed cardiac diagnosis were confirmed as cardiac, which highlights the importance of investigating and recording the aetiology as confirmed in the medical record or by autopsy.

### Limitations

This is a small retrospective study conducted within a local health network in Australia and care should be taken when generalising the findings. Crude and age-adjusted incidence calculations were made using enumerated population data that was averaged between 2011 and 2016 to account for dynamic population changes and may not accurately reflect the true at-risk population. OHCA incidence calculations may be underestimated due to missing cases within SAAS-CAR during the study period (24). Arrest location and EMS response times are important predictors of survival that may have influenced outcome but were not available within SAAS-CAR during the study period. Arrest postcode was used as a surrogate for patient SES but may not reflect the patient's true level of advantage and disadvantage. Finally, investigation of sex differences in outcome of EMS-treated OHCA was limited due to small sample size and the findings should be confirmed in a larger sample. Nonetheless, this study provides important findings on sex differences in incidence and outcome of OHCA according to both presumed and confirmed cardiac and non-cardiac etiology.

## Conclusions

Women were less than half as likely to experience OHCA than men and the incidence of OHCA in women of any given age group was similar to that of men 10 years younger within a local health network. The effect of sex on survival to discharge after EMS-treated OHCA was influenced by precipitating etiology. Women with non-EMS witnessed presumed cardiac OHCA were more likely to present with unfavourable predictors of survival and were more likely to arrest in location associated with low SES, but there was no sex difference in adjusted survival. Analysis of adjudicated aetiology in the hospitalised sub-cohort suggests that survival after non-cardiac OHCA may be higher in women than men, but this finding requires further validation.

## Data Availability Statement

The datasets presented in this article are not readily available because access to participant identifiable data is subject to relevant institutional approval(s). Requests to access the datasets should be directed to MW, melanie.wittwer@adelaide.edu.au.

## Ethics Statement

The studies involving human participants were reviewed and approved by Central Adelaide Local Health Network (CALHN) Human Research Ethics Committee. Written informed consent for participation was not required for this study in accordance with the national legislation and the institutional requirements.

## Author Contributions

MW: conceptualisation, methodology, investigation, formal analysis, and writing—original draft. EA: conceptualisation and writing—original draft. CH: data curation, writing—review, and editing. MT: data curation, writing—review, and editing. CZ: supervision, writing—review, and editing. JB: supervision, writing—review, and editing. MA: conceptualisation, supervision, writing—review, and editing. All authors take responsibility for the integrity of the data and the accuracy of the data analysis. All authors read, critically reviewed, and approved the final manuscript.

## Conflict of Interest

The authors declare that the research was conducted in the absence of any commercial or financial relationships that could be construed as a potential conflict of interest.

## Publisher's Note

All claims expressed in this article are solely those of the authors and do not necessarily represent those of their affiliated organizations, or those of the publisher, the editors and the reviewers. Any product that may be evaluated in this article, or claim that may be made by its manufacturer, is not guaranteed or endorsed by the publisher.
