# Supplementary material for: Sex Differences in Incidence and Outcome of Out-of-Hospital Cardiac Arrest Within a Local Health Network
Source: Front Cardiovasc Med. 2022 Apr 8;9:870696. doi: 10.3389/fcvm.2022.870696 (PMC9024218; doi:10.3389/fcvm.2022.870696)
Supplement: Supplementary file 1 [file Table_1.pdf]

Supplementary Material

Table S1: Inflation factors for incidence calculations

**EMS-attended**	**Total**	**Females**	**Males**
Number of cases	1970	691	1279
Age missing	54	8	46
Inflation factor	1.0274	1.0116	1.0360
Uninflated crude incidence (per 100,000 person-years)	144.7	99.1	192.6
Uninflated age-standardised incidence (per 100,000 person-years)	136.2	94.9	199.6
**EMS-treated cohort**			
Number of cases	772	273	499
Age missing	8	0	8
Inflation factor	1.0103	n/a	1.0160
Uninflated crude incidence (per 100,000 person-years)	56.7	39.2	75.2
Uninflated age-standardised incidence (per 100,000 person-years)	54.1	38.1	71.8
**Non-EMS witnessed presumed cardiac sub-cohort**			
Number of cases	501	163	338
Age missing	0	0	0
Inflation factor	n/a	n/a	n/a
**Non-EMS witnessed obvious non-cardiac sub-cohort**			
Number of cases	161	63	98
Age missing	7	0	7
Inflation factor	1.0434	n/a	1.0714
Uninflated crude incidence (per 100,000 person-years)	11.8	9.0	14.8
Uninflated age-standardised incidence (per 100,000 person-years)	11.7	9.0	14.6
